# Medical Expert and Medical Evidence

**Published:** 1875-03

**Authors:** M. A. McClelland

**Affiliations:** Knoxville, Ill.


A MONTHLY RECORD OF

*Medicine, Surgery and the Collateral Sciences.*

EDITED BY J. ADAMS ALLEN, M.D., LL.D.: AND WALTER HAY, M.D.

---

VOL. XXXII. — MARCH, 1875. — NO. 3.

---

### Original Communications.

ARTICLE I.

### THE MEDICAL EXPERT AND MEDICAL EVIDENCE.

BY M. A. McCLELLAND, M.D., KNOXVILLE, ILL.

(Continued from page 108.)

"PRIVILEGED" WITNESSES.

"Whatever, in connection with my professional practice, or not in connection with it, I see, or hear, in the life of men, which ought not to be spoken of abroad, I will not divulge, as reckoning that all such should be kept secret. While I continue to keep this oath unviolated, may it be granted me to enjoy life, and the practice of the art, respected by all men, in all times." (Extract from Hippocratic Oath.)

"Secrecy and delicacy, when required by peculiar circumstances, should be strictly observed; and the familiar and confidential intercourse to which physicians are admitted in their professional visits, should be used with discretion, and with the most scrupulous regard to fidelity and honor. The obligation of secrecy extends beyond the period of professional services; none of the privacies of personal and domestic life, no infirmity of disposition or flaw of character observed during professional attendance, should ever be divulged by the physician, except when he is imperatively required to do so. The force and necessity of this obligation are indeed so great, that professional men have, under certain circumstances, been protected in their observance of secrecy by courts of justice." (Code of Ethics, Am. Med. Association.)

"A precedent in law is a mighty authority; and I am quite satisfied that a point which has so often and so uniformly ruled, will never be ruled otherwise in the courts of Westminister Hall. I am also well aware that to law, and rules of court, we must yield, or the administration of justice would be impeded. But although satisfied on these points, I am not contented that we should be placed beyond the pale of those to whose private and confidential dealing with their fellow citizens such respect is shown. I will not go at large into this question, my design being merely to draw the notice of my brethren to the circumstance, and to put them upon their guard as far as possible; yet will I say, that circumstances may occur in which a man of a delicate and honorable mind, being the depositary of certain things communicated to him, either under seal of professional or private confidence, would endure much ere he would reveal. It will at once strike the manly mind, that in regard to females we might be called upon to reveal that of which the promulgation would to them be worse than death." (Smith's Analysis of Med. Evidence.)

"We believe it to be the moral right and duty of medical men to refuse to disclose, in a court of justice, secrets intrusted to them in professional confidence, and we have always acted on such belief. If physicians become the repositories of secrets, under the full conviction, on the part of society, of our moral and professional obligations to hold them sacred,—secrets which otherwise never would have been revealed,—who can believe that there is any earthly power which ought to wring them from us, or which can, if we rightfully understand our privileges and duty. If private confidence is thus to be broken upon every imaginary necessity, where is the end to the mischievous consequences that would arise, especially at this day, where every trial is published to the world through the medium of the public prints. The lawyer is shielded from the obligation of revealing the secrets of his client, on the ground that it is necessary he should be acquainted with the real facts in the case, for the purpose of conducting the defense, and because life and property are at stake. But we ask, if character and reputation are not often of equal value, and whether either of the former could be enjoyed without the possession of the latter? So also it may be observed, that the patient communicates freely with his physician, for the purpose of judgment; no circumstances whatever, will warrant their publication to the world. In the case of females, such a disclosure would be in the highest degree indelicate, and often worse than any punishment that could be inflicted." (Charles A. Lee, M.D.)

Professor Stephen Smith says, ("Doctor in Medicine,"): "In whatever light we view this subject, the fact is constantly prominent, that the moral obligation of the physician to retain, inviolate, all communications of a confidential nature, is undisputed. By the Hippocratic oath he is not to divulge what he sees or hears, whether in connection with his professional practice or not. The code of ethics of the great governing body of this country pledges him never to divulge the privacies of personal and domestic life, nor the infirmities of disposition, nor flaws of character observed during professional attendance, except when imperatively required to do so. It is only in courts of justice that the seal of secrecy can be broken, and even here the peculiar moral obligations of the physician are acknowledged and respected."

On the other hand, Professor Elwell, (Malpractice and

Med. Evid., p. 322), says: "The discovery, vindication, and establishment of truth, and the punishment of crime, are the main purposes of the existence of courts of justice. For this the rules of law are established, and to this end witnesses are put upon the stand, and they are to tell the whole truth, bearing upon the case, unless there is some special, powerful reason why they should not.

"The great interests of government, life, liberty and private property, all depend upon a well settled system of evidence, by the rules of which the whole truth may be as surely as possible brought out. Any privilege that a witness may enjoy, which permits him to retain in his own bosom a knowledge of facts bearing upon the case at issue, contravenes the great object of all law, just to the extent that this privilege from testifying is permitted to reach. As facts are taken or kept from a court or jury, the means of arriving at a just conclusion are abridged, and from the want of the light of these very facts, thus kept out of sight, the ends of justice may be defeated,—the guilty escape,—the innocent suffer,—and possibly great public interests may be endangered.

"The reasons then, if any, for permitting private interests to outweigh the certainty of judicial investigations and their results, should, it would seem, be cogent and convincing. The justification should be a complete one, that will permit a witness to refuse information, essential, perhaps, to the just solution of an important issue, possibly involving life. This information a privileged witness is permitted to refuse to give to a jury. Should not such a witness be subjected to the severest scrutiny, and confined cautiously and carefully to the exceptional cases where it is claimed greater evil will result if the truth is permitted to come out? A rule that limits the sources of information in courts of justice; that prevents truth from being followed into every channel, and brought forth from every lurking place; thus hazarding just results; is an exception and an anomaly in law, and should be subjected to all the disabilities of an anomaly."

All of which is very true. But, if on the grounds of public policy, attorneys are not permitted to disclose the secrets of their clients, in respect to property and reputation, on the same grounds physicians should have accorded to them the same right. To the statement that the object of the rule is to require that the "entire professional intercourse between client and attorney, whatever it may have consisted in, should be protected by professional secrecy," we may reply, the same reason applies with equal force to the patient and physician. To the statement that the attorney stands in the place of the client, we say no, no more so than the physician does who has received statements from his patient, the better to draw his diagnosis and to determine his treatment. "No interest, therefore, but that of the client, permits the attorney to refuse to give in his testimony in the case, and this interest, it is said, 'comprehends the entire professional intercourse between client and attorney, whatever it may have consisted in.'" (Elwell.) It is only the *interest* of the patient that the physician would subserve by the adoption of the same rule in his case. Mr. Chief Justice Shaw says: "So strictly is the rule held, that the privilege extends only to communications made by client to his attorney for the purpose of obtaining legal advice." This is all that physicians ask.

This right is now recognized by statutory enactments in some of the States.

*Confidential Communications.*—"No person authorized to practice physic or surgery shall be compelled to disclose any information which he may have acquired from his patient while attending him in a professional character, and which information was necessary to enable him to prescribe as a physician, or do any act for him as a surgeon." (Ark. Statutes, ch. 181, § 21.)

So also in California, (Cal. Stat., Hittell's ed., 1850–64, § 398,) "a licensed physician or surgeon may be examined, but only with 'consent' of patient."

So also in Michigan: "No person duly authorized," etc., etc., as in Arkansas.

Minnesota has a statute similar to California, limited, however, to "civil actions." (Ordronaux Jurisp. of Med.)

Let us hope that the same equitable provisions will soon obtain in other States. In the meantime it must be remembered that "no matter how sacred the trust a confidential communication made to a physician or a divine, the law *forces* a disclosure." Whether the matter is relevant or not, is for the consideration of the presiding judge, the witness under no circumstance is to refuse the answer, on the ground of irrelevancy. (*Wright* v. *Wilkes*, Taylor's Med. Jurisp., ed. by Penrose, p. 42.)

The witness has an unquestionable right to express his opinion in his own way; he is not on the stand to answer any and every question counsel put to him; especially he is not to answer by 'yes' and 'no' without the privilege of explaining it. The bluster of counsel on these occasions may be quietly ignored. The court will sustain him in his endeavor to make himself thoroughly understood. This rule is to be particularly observed in "hypothetical" cases, where the form of the question is only limited by the ingenuity of the counsel. Do not form an opinion on a few *selected* facts.

The witness is to open to the jury the most ample view of all the facts which belong to the disputed transaction. Many facts, from their very nature, absolutely exclude direct evidence to prove them. Thus it is in questions of skill and judgment, the knowledge of the relations must be derived from those who are possessed of the proper qualifications for forming a conclusion on the subject. Juries in general are to form conclusions by aid of their own experience, but in this class of cases they cannot be presumed to possess that experience which will enable them to draw correct inferences from the circumstances, hence the expert is called, in order to obtain those inferences that only a few persons, exercising a particular science, art or profession, are capable of drawing, and he will not be permitted to refuse to testify on any claim of privilege whatever.

### INQUESTS.

In service before a coroner's jury, the medical witness should never give an opinion without demanding and obtaining the privilege of making a *post mortem* examination. For making this it is his right to demand and receive a fair compensation. As this is one of the medico-legal duties the general practitioner will most frequently be called upon to perform, directions in detail as to the best manner of performing it will not be out of place in this connection. In such examinations two physicians should be associated, inasmuch as the details are so minute that no mind could retain them all, therefore one should make a written record as the dissections are made by the other; notes should be examined and corrected immediately. Such notes would not be evidence, but the physician could use them to refresh his memory. In respect to whether these notes could be copied, and the copy thus used, there is some difference of opinion. It is usual not to permit such copy. In respect to the necessity for such notes, it is to be remembered that in many cases the medical witness' duties do not close with his appearance before the coroner. He will frequently have to appear, farther, before the criminal court, and there testify as to professional opinions, deduced from the appearances he has observed.

### POST MORTEM EXAMIMATIONS.

In an inspection of a body found dead the memoranda should show the date and place of examination. The following plan adopted from CASPAR, covers the essential points of such an examination:

#### EXTERNAL APPEARANCES.

1. Note the external appearance; apparent age; quantity and color of the hair; color of the eyes.
2. Position of the tongue.

3. Foreign bodies in the mouth, ears, nose and other natural passages.

4. Rigor mortis.

5. Color of body in respect to *post mortem* changes, putrefaction, *post mortem* stains,* tattoo marks, scars and condition of skin, as *cutis* anserina, etc.

6. Œdema, emphysema.

7. Suspicious marks, whether *ecchymotic* or *post mortem.*

8. Appearances of posterior surface of body, hands and feet.

9. Appearance of neck and sexual parts.

### INTERNAL INSPECTION.

#### 1. *CRANIAL CAVITY.*

10. Condition of soft parts covering the skull, and condition of skull bones.

1. Condition of meninges.

12. Condition of brain as respects softening, conjestion, extravasation of blood, and tumors.

13. Condition of the ventricles, choroid plexus, sinuses.

14. Condition of cerebellum, pons varolii and medulla.

15. Condition of basis cranii.

#### 2. *THORAX.*

16. Position of the different organs, adhesions, color, extravasation or effusion.

17. Condition of the large blood vessels.

18. Pericardium, coronary veins, and condition of cavities of the heart.

19. Appearance and contents of larynx and trachea.

20. Condition and contents of œsophagus.

* "No incision into a *post mortem* stain, be it ever so deep or bold, will ever give vent to effused fluid or coagulated blood ; at the most there will be but a few bloody points, the result of cutting across some small veins of the skin, whilst in the smallest ecchymosis the effused blood will at once be brought to light by the incision." (Caspar, vol. 1, p. 19, New Sydenham Soc. Pub.)

21. Condition of pleural cavities, in respect to effusions and adhesions.

3. *ABDOMINAL CAVITY.*

22. Organs, position and contents of ; ulcerations, tumors, or perforations.
23. Pancreas.
24. Liver, gall bladder, condition and contents.
25. Spleen.
26. Mesenteries and omenta.
27. Kidneys, conjested, fatty, waxy, etc.
28. Bladder and its contents.
29. Vena cava, aorta.

At the close of the examination express briefly the opinion that—

1. The deceased died of——— or from———.
2. The probable time of death.
3. Explanation of any unnatural marks or appearances, whether sufficient to cause death.

It may not be necessary to add that the physician is not to hazard an opinion, in a case of suspected poisoning, from a mere *inspection* of the stomach, as to the causes giving rise to the *appearances* observed. It might be replied to a question, that the *appearances might* depend upon poison, but that is a question to be decided by chemical analyses.

In drawing up reports and in giving opinions orally, physicians are too apt to give the *remote* rather than the *present* cause of the appearances presented upon *post mortem* examination. Thus the stomach of one supposed to be poisoned is found to have patches of inflammation scattered over its internal surface, and the physician, if not very careful, will say or write, "the appearances are of *poison*," when he should have said "appearances of *inflammation*" of the stomach.

CHEMICAL ANALYSES.

In respect to chemical analyses in cases of poisoning, general practitioners have but little if anything to do.

This is the province ordinarily of the analytic chemist, who is presumed to have all the apparatus needed for making such analyses. A theoretical knowledge of the processes in such analyses is however expected of the medical expert. There are duties, however, in this connection that fall to the physician alone, the proper performance of which will not only reflect honor upon him, but will favor the ends of justice. This duty relates to the symptoms during the last illness, and to the performance of the *post mortem* examination, and the proper preservation of such parts of the body as are deemed necessary to be examined.

It will frequently happen that the physician who is called upon to make the *post mortem* examination, has attended the dead person through his last illness, he should therefore be well informed as to what are the evidences of poisoning in the living body. When did the symptoms he was called to treat first manifest themselves, and was the individual in good health immediately prior to the present illness? Did these symptoms occur immediately or soon after eating or drinking, and did others partake of the food or drink at the same time? If so, are they affected similarly? In irritant poisoning, pain, vomiting, and purging, are the first prominent symptoms. In what order did they occur? In a recent trial for murder by poisoning, it was intended by the defense to make it appear that the symptoms during life were to be referred to cholera morbus, which, at the time of the death, was prevalent in the community. But in cholera morbus the vomiting and purging are simultaneous. Cholera also in its malignant form may rapidly end in death, but it should be remembered that its symptoms are directly the reverse of irritant poisoning.

Inquiry should be instituted as to the habit, idiosyncracy or recent disease that may have been present.

I have had recently a patient under my charge for glaucomatous symptoms, who regularly used sixty grains of morphine every seven days, or a trifle more than the third of a grain an hour, an amount sufficient to bring about, as a rule, a fatal narcotism in one not used to the drug. On the other hand, Dr. Christison relates the case of a person unacustomed to the use of opium, who took nearly an ounce of good laudanum without any effect. This same idiosyncracy may convert substances used as food, harmless in themselves, into virulent poisons. I am one of those unfortunates who dare not eat oysters. Their injestion is uniformly followed by an irruption of urticaria, cramp in the stomach, vomiting and purging, accompanied by a decided chill, and excruciating intestinal pain. Muscles, fish, pork, mushrooms, have been observed to give rise to the same train of symptoms in others. Hasty conclusions should therefore not be published, for symptoms alone should never decide the question. "At best they only justify an opinion in favor of poisoning as probable."

What are poisons? The definition given by Dr. Guy would seem to answer the inquiry so completely that I here give it. He says: "A poison is any substance or matter, solid, liquid or gaseous, which, when applied to the body outwardly, or, in any way introduced into it, without acting mechanically, but by its own inherent qualities, can destroy life." The term is an abstract one, like a "deadly wound," "insanity," and such like terms, which nearly every one thinks he has a definition for. The above is not free from criticism, but for all practical purposes it is sufficient.

The expert should make himself familiar with a brief yet comprehensive classification of poisons. That given by Dr. Guy is here given in a tabulated form.

POISONS.

| | | | |
|---|---|---|---|
| 1. Inorganic. | *a.* Corrosive.<br>*b.* Irritant. | 2. Organic. | *a.* Irritant.<br>*b.* Affecting the brain.<br>*c.* Affecting the spinal cord.<br>*d.* Affecting the heart.<br>*e.* Affecting the lungs. |

(To be continued.)

